# Dynamic Evolution of the Ecological Carrying Capacity of Poverty-Stricken Karst Counties Based on Ecological Footprints: A Case Study in Northwestern Guangxi, China

**DOI:** 10.3390/ijerph17030991

**Published:** 2020-02-05

**Authors:** Shana Shi, Baoqing Hu, Yan Yan, Xiaoqing Li, Kaichun Zhou, Chuanyong Tang, Binggeng Xie

**Affiliations:** 1 College of Resources and Environment Science, Hunan Normal University, Changsha 410081, China; shala429@163.com (S.S.); lixiaoqing1919@foxmail.com (X.L.); zhoukaichun@hunnu.edu.cn (K.Z.); 2 Key Laboratory of Environment Change and Resources Use in Beibu Gulf, Nanning Normal University, Ministry of Education, Nanning 530001, China; hbq1230@gxtc.edu.cn (B.H.); yanyan168166@163.com (Y.Y.); 3 School of Geography and Planning, Nanning Normal University, Nanning 530001, China; supertang163@163.com

**Keywords:** karst county, poverty rate, ecological footprint, ecological carrying capacity, dynamic evolution, northwestern Guangxi

## Abstract

The karst area in northwestern Guangxi is poor, underdeveloped, and ecologically fragile. It is experiencing rocky desertification, which creates challenges that are more severe than those of other regional ecological environments. In this paper, the ecological footprint (EF) model is used to analyze the ecological carrying capacity (EC) in northwestern Guangxi from 1995 to 2015, and the differences in karst counties with different poverty levels are discussed. The results show that (1) since 1995, the EC of northwestern Guangxi has continued to decrease, the EF has continued to increase, the ecological deficit (ED) has been expanding, and the status of the region has been unsustainable for a long time. (2) The evolutionary patterns, EF and EC of karst counties with different poverty levels are different. The county with the lowest poverty rate has the fastest growth rate of the per capita EF. The county with the largest proportion of karst area has the lowest EC. (3) It is recommended that different types of counties take different measures, including strengthening ecological environment protection, carrying out rocky desertification control and ecological resettlement projects, and reducing energy consumption. This study can provide information for the sustainable development of the karst region and provide decision support for regional poverty alleviation.

## 1. Introduction

Currently, the development of human society is confronted with two major themes, development and the environment, to which increasing attention has been paid by various states’ governments and academia [1]. The contradiction between limited natural resources and the growth of human demand is one of the core issues of regional and global sustainable development [2]. The coordination of the relationship between social economic development and environmental protection is the key to achieving sustainable regional development [3]. The Sustainable Development Goals are a call for action by all countries to promote prosperity through measures such as sustainable consumption and production, sustainable management of natural resources, and confrontation of climate change and environmental protection [4]. To quantitatively assess the sustainability of regional development, Rees et al. proposed the concept of the ecological footprint in 1992, which converted the consumption of natural resources into the area of ecologically productive land [5]. Subsequently, Wackemagel et al. used an ecological footprint to predict the available and occupied ecological space at the national level [6]. The ecological footprint method provides a simple and direct theoretical basis with which we can assess the sustainability of regional development [7].

Domestic and foreign scholars have conducted much research on the ecological footprint theory since its introduction. They achieved certain results in the study of ecological footprints at different scales and in different fields. The research has been mainly focused on large scales, with studies at the global and national levels [8,9,10], provincial level [11,12], and municipal level [13,14]. Some scholars have conducted research at the county level, such as adjusting the area of cultivated land through the planting index, improving the ecological footprint method, calculating the cultivated land area required by humans, using the improved ecological footprint method to analyze the ecological supply and demand situation in Funing County, and pointing out the patterns of animal husbandry. The issues addressed in previous research are key factors affecting the regional ecological footprint [15]. Some scholars have also used the ecological footprint-based service value method to analyze the distribution characteristics, evolution pattern, and driving factors of the ecological capacity index of a county [16].

Ecological footprint research mainly includes ecological footprint theory research [17,18], ecological footprint model research [19,20], ecological footprint assessment or evaluation [21,22], ecological footprint change and influencing factor analysis [23,24], specific types of ecological footprint assessment [18,25], and research on other aspects. Among these, the ecological footprint is used to evaluate the ecological carrying capacity of specific land cover types, mainly including research on specific types of areas such as river basins, mountainous areas, islands, and karst areas. For example, Zhang et al. combined the ecological footprint, remote sensing, and a geographic information system to study the per capita ecological carrying capacity of Dongting Lake and conducted a dynamic spatiotemporal evaluation of the Dongting Lake [26]. Xiang et al. established an account of an ecological footprint by constructing an ecological capacity assessment model to predict the ecological carrying capacity of the northern slope of the Tianshan Mountains [27]. Ma et al. proposed a general conceptual model for the systematic evaluation of marine ecosystem carrying capacity and established an evaluation index system in China’s Dongtou Islands [28]. Wang et al. constructed an index of balanced supply and demand for the ecological carrying capacity of cultivated land in karst areas and analyzed and evaluated the current situation of the balance of supply and demand of cultivated land ecological footprints in Bijie city, which is dominated by karst landforms. This can provide a basis for decision-making in Bijie city’s agricultural industrial structure adjustment, ecological security early warning, regional economic development, and overall land use planning [29].

At present, the evaluation of the ecological carrying capacity by ecological footprint is mainly based on large-scale research at or above the municipal level. The scale of the county level and below is relatively small, and the research at the county level in the karst area is minimal. In addition, most of the research is based on the analysis of the current status and dynamic changes in the overall ecological footprint or ecological carrying capacity of a region. There are few studies on regional differences, and it is difficult to make decisions about the development of different units within a region.

In the view of the global efforts to increase the welfare of different groups and share prosperity, spatial analysis of poverty has become an international question [30]. Poverty is a complex and multi-level problem, but accurate measurement is often inappropriate [31]. It cannot be comprehensively measured from economic data alone. At present, scholars carry out research related to poverty from different fields. Poverty research is not only economic, but also psychological and physical, including research on what people can be done, what kinds of people they can become, and various effects of poverty [32,33,34]. Therefore, its measurement standard should be a combination of quantitative and qualitative. Since the 1980s, the research on poverty, degradation of ecological environment, and their interrelation, has become a hot topic in academic circles. Scholars have carried out a lot of research work around the interaction between these two factors [35,36,37]. Poverty alleviation and ecological environment protection as an important part of sustainable development [38,39], especially in poor areas and ecologically vulnerable areas in developing countries, are the core content [40]. However, at present, the cruel reality deviates from the expectation of sustainable development [41]. In order to develop an economy rapidly, human consumption of resources far exceeds the regeneration ability of natural ecosystem, which results in insufficient supply of resources and seriously threatens human survival and development [42].

Studies showed that over the past 50 years, human impacts on ecosystems have been more rapid and widespread than ever before [43]. The severity of the contradiction between social and economic development and ecological environment in different countries and regions is different. Especially in some poverty-stricken areas, in order to speed up economic development, people will not hesitate to destroy the environment, resulting in the decline of ecological carrying capacity. Karst area is one of the most vulnerable natural environments in the world, which is very vulnerable to various changes, degradation, and pollution. The carrying capacity of the natural environment here is very low [44]. Karst system is a non-renewable resource, and disturbed by more and more human activities [45]. Although all of them are fragile ecosystems, the economic development level, ecological environment condition, and human activity intensity of different karst areas are different. Some karst areas have less damage to the ecological environment, and some karst areas have more serious damage. However, the main environmental problems in karst areas are similar. For example, the water resources problems in karst areas are more serious, which becomes the focus of domestic and foreign scholars [46,47]. In addition, due to the regional differences of various influencing factors, some karst areas have environmental pollution problems, and some have ecological environmental problems such as reduction of animal species [48,49].

The basic characteristics of China’s karst region include of a vicious circle of poverty and ecological environment destruction, which is the epitome of China’s sustainable development [50]. Soil erosion has been a serious problem for a long time due to unsustainable farming and increased population pressure, and the area of rocky desertification has been expanding, which has led to a vicious circle of ecological environment destruction in the karst mountains [51]. As a unique region, the evaluation and prediction of the ecological footprint and ecological carrying capacity of karst areas are critical to better understand the current situation and development trend of the region, to achieve sustainable development of resources and the environment and to promote economic development and poverty alleviation in karst areas. In 2012, the Poverty Alleviation Office of the State Council of China published a list of 592 poverty-stricken counties, which are concentrated in the central and western regions of China. In recent years, China has made tremendous achievements in poverty alleviation. The economy, production, and living conditions in poor regions and areas have been significantly improved and developed, and the poverty situation has been alleviated to a certain extent. However, due to the large scale of the poverty alleviation targets and the prominent imbalance in regional development, the contradiction between the social and economic development and the ecological environment carrying capacity in poverty-stricken areas has also intensified, so the pressure on the ecological environment has been increasing. The karst area in northwestern Guangxi is one of the poorest areas in China. The most prominent feature of this area is the integration of the old, the young, and the poor near the border and in the mountains [52]. Located in Western China, it is a traditional agricultural mountainous area. The area is large, the natural conditions are poor, the public transport is inconvenient, and the social and economic development is relatively weak. Therefore, the Chinese government has determined that the mountainous area in northwestern Guangxi is a concentrated and particularly poor area.

Therefore, the purpose of this study is to (1) use the ecological footprint model to calculate the per capita ecological footprint and per capita ecological carrying capacity of the karst area in northwestern Guangxi from 1995 to 2015 and (2) divide northwestern Guangxi into different levels according to the poverty rate. Representative karst counties are used to evaluate the per capita ecological footprint and per capita ecological carrying capacity of karst counties with different poverty levels and to compare the differences between the per capita ecological footprint and per capita ecological carrying capacity of these counties. (3) According to the different poverty degrees of the karst counties, corresponding countermeasures and suggestions to improve the ecological carrying capacity are proposed.

## 2. Materials and Methods

### 2.1. Study Area

**Figure 1:**
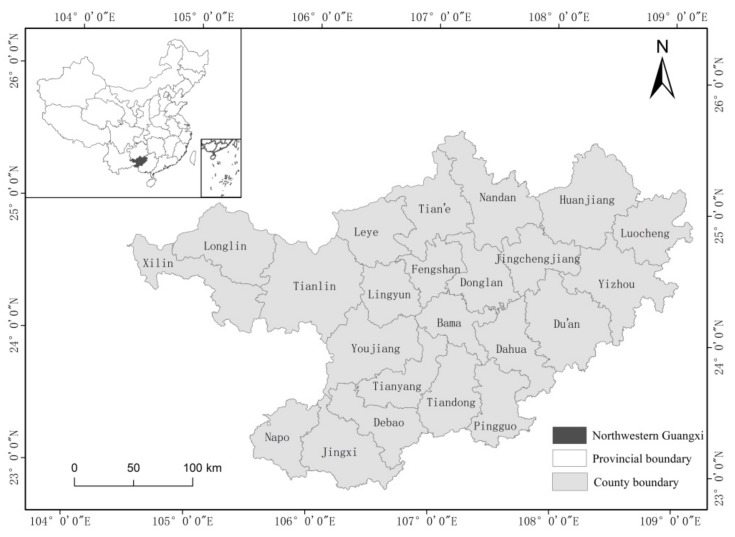
Administrative division map of the research area, which includes 21 counties.

The karst areas in northwestern Guangxi (104°29′–109°09′ E, latitude 23°41′–25°37′ N) include the cities of Baise and Hechi, which are located in southwestern China (Figure 1). The study area is 697,700 square kilometers, of which the karst area accounts for more than 60% and the mountainous area accounts for more than 80%. There are many rivers in the study area, such as the Youjiang River, the Hongshui River, and the Longjiang River and its tributaries. Northwestern Guangxi has a population of more than 8.73 million. The karst area in northwestern Guangxi is an area where human living conditions are very poor. Sixteen counties have been listed as extremely poor in the rocky desertification areas of Yunnan, Guangxi and Guizhou, and the 21st Century Agenda of China has listed this region as one of the areas where the government has given priority support to eradicate poverty.

### 2.2. Data Sources

The administrative division data used in this paper are from the data cloud website of the Chinese Academy of Sciences [53]. The land use data were collected from five issues of remote sensing monitoring data of land use status in China from 1995 to 2015 and were obtained from the Resource and Environment Science Data Center of the Chinese Academy of Sciences [54]. When the ecological footprint was calculated, the global average output data were mainly derived from The Food and Agriculture Organization (FAO) statistical database. The output data, energy account consumption data, GDP data, and population data of various biological resources accounts were obtained from the statistical yearbook of Guangxi from 1995 to 2015, the Baise statistical yearbook, the Hechi statistical yearbook, the Statistics on National Economic and Social Development of Baise City, the Statistical Summary of National Economy in the Hechi Area, and the Economic and Social Statistical Yearbook of the Hechi Region. The types of resources in the biological resources account and the energy account were determined according to the main types of statistics in the previous statistics of the study area. The poverty rate was calculated according to the proportion of regional poor people in the total population. The poor people come from the 13th five-year plan for poverty alleviation in each county. The proportion of karst area was calculated by the proportion of regional karst landform area to the total area. The data of karst landform comes from the atlas of the People’s Republic of China (1:1 million) compiled by the Chinese Academy of Sciences. In addition, the equivalence factors proposed in [55] and others were used in this study (Table 1). This study the corresponding regional yield factor in northwestern Guangxi was calculated (Table 1). The yield factors were obtained by comparing the biomass production data of northwestern Guangxi in 2015 with the world average biomass production data calculated by the FAO of the United Nations in 2015. Due to a lack of data, the global average output of some projects refers to the relevant data used from [56].

### 2.3. Research Methods

#### 2.3.1. Calculation of the Ecological Footprint (EF)

The ecological footprint (EF) is the sum of all types of land consumed by human activities. This includes six types of land: arable land, pasture, forest, built-up area, water area, and fossil energy area [55]. The productivity of these six types of land varies greatly. To compare the calculated results, each type of land is multiplied by an equivalence factor, and then the weight of the equivalent bioproductive land area is used to calculate the EF [3]. The formula for the calculation is as follows:

(1) $$EF=N\times ef=N\times r_{j}\times \sum a_{i}=N\times r_{j}\times \sum \frac{c_{i}}{p_{i}}\;(i\;=\;1,\;2,\;\ldots ;\;j\;=\;1,\;2,\;\ldots ,\;6)$$

where *EF* is the total ecological footprint, *N* is the population, *ef* is per capita ecological footprint, *a_i_* is the per capita ecological footprint component converted by the *i*th type of consumption, *i* is the input type of consumption of goods and resources, *N* is the number of consumption items, *r_j_* is the equivalence factor corresponding to the *i*th type of land, *p_i_* is the average world production corresponding to the *i*th type of goods or services (kg/hm^2^), and *c_i_* is per capita consumption corresponding to the *i*th type of goods (kg).

#### 2.3.2. Calculation of the Ecological Carrying Capacity (EC)

The ecological carrying capacity (EC) refers to the sum of all the productive land that can provide the resources and energy consumed by residents in an area [2]. The difference in resource endowment and land productivity in different counties and regions leads to the difference in the unit yield of the six land types. To compare the actual area of a given type of biologically productive land in different countries and regions, the standardization and yield factors of different types of biologically productive land were adopted [3]. The formula for the calculation of the EC is as follows [5]:

(2) $$EC=N\times ec=N\times \sum_{i=1}^{n}a_{j}r_{j}y_{j}$$

Here, *EC* is the total ecological carrying capacity; *a_i_*, *r_i_*, and *y_i_* represent the per capita biologically productive area, equivalence factor, and production factor, respectively; *N* represents the population; and *ec* is the per capita ecological carrying capacity.

The World Commission on Environment and Development (WCED) released a report called “Our Common Future”. According to the proposals in this report, 12% bioproductive land area should be achieved in the calculation of regional ecological carrying capacity for the protection of biodiversity in a region [57].

#### 2.3.3. Calculation of the Ecological Deficit (ED) and the Ecological Surplus (ES)

The ecological deficit (ED) and ecological surplus (ES) reflect the utilization of natural resources by the population in the study area. When EF is subtracted from EC and the calculation result is positive, it is called ecological surplus, which indicates that the resource demand of the region is in a sustainable development state within the ecological carrying capacity of the region. When the result is negative, it is called ecological deficit, which indicates that the demand for resources in a region exceeds the ecological carrying capacity of that region and that development is not sustainable [58]. ES and ED can be expressed by the following equation:

(3) $$ED\left(ES\right)=EC-EF=N\times \left(ec-ef\right)$$

where *N*, *ec*, and *ef* are the population, net per capita *EC*, and per capita *EF*, respectively.

## 3. Results and Discussions

### 3.1. Evaluation of the EF and EC in Northwestern Guangxi

#### 3.1.1. Calculation of the EF in Northwestern Guangxi in 2015

The calculation of the EF included biological resource consumption and fossil energy consumption.

- Consumption of biological resources

We divided the consumption of biological resources into crop products, animal products, forest products, and other products. Of all the biological resources, 31 species were selected, including cereals, vegetables, and melons as well as those that produce meat, milk, and aquatic products. We used the world average production data on biological resources calculated by the United Nations (UN) Food and Agriculture Organization in 1993 to convert the production area of biological resources [59]. The results are given in Table 2. By comparing the consumption of various projects in northwestern Guangxi, we found that the consumption of cereals was the highest. Meat consumption involves predominantly pork, while consumption of beef and lamb was much lower.

- Consumption of fossil energy resources

We chose five main types of fossil energy: coal, gasoline, diesel oil, liquefied petroleum gas, and electricity. The electricity consumption was converted into construction land area, the other energy consumption types were converted into fossil production land area, and the energy consumed for heating was converted into fossil land area according to the heat conversion coefficient used in global energy statistics [60]. The results are given in Table 3. The results suggested that the area per capita of the fossil energy use in northwestern Guangxi in 2015 was 0.63 hm^2^, while the area per capita of coal use was 0.54 hm^2^, accounting for 85% of the fossil energy area per capita. These findings indicated that energy consumption in northwestern Guangxi is still dominated by coal; however, coal causes severe environmental and air pollution, and therefore, the energy consumption structure of northwestern Guangxi is not efficient.

#### 3.1.2. Calculation of EC in Northwestern Guangxi in 2015

According to the per capita biological production area available in the northwestern Guangxi region in 2015, the per capita EF and per capita EC were calculated and compared. In 2015, the largest per capita EF in northwestern Guangxi was that of fossil energy land use, which was 0.6931 hm^2^, followed by arable land and grassland (Table 4). Similarly, the largest per capita EC was that of woodland, at 0.4357 hm^2^, followed by arable and building land.

The per capita EF for northwestern Guangxi in 2015 is 1.9657 hm^2^, while the per capita EC is only 0.7692 hm^2^ (Table 5). After deducting 12% of the biodiversity conservation reserve from the per capita EC, we found that the available per capita EC is 0.6769 hm^2^, and the per capita ED reached 1.2888 hm^2^. The EF of the region is nearly three times greater than its EC, indicating that the supply of land resources in the region is far from meeting the demand and that the ecological environment is in an unsustainable state.

Among the land use types, the deficit in land used for fossil energy is the most serious, followed by grassland and arable land. This is closely related to the underdevelopment of northwestern Guangxi. Industrial production consumes a larger amount of energy in the process of regional economic development. The consumption of fossil energy will lead to the aggravation of regional water, land, and other resource pollution, reduce the regional economic growth rate, and affect regional sustainable development. Moreover, the inhabitants of this region essentially form a farming society, which leads to excessive an EF in pasture and arable land. Only the forestland is in a surplus, but the forests also tend to be saturated, which should also be taken seriously.

#### 3.1.3. Dynamic Evolution of the Per Capita EF and EC in Northwestern Guangxi from 1995 to 2015

**Figure 2:**
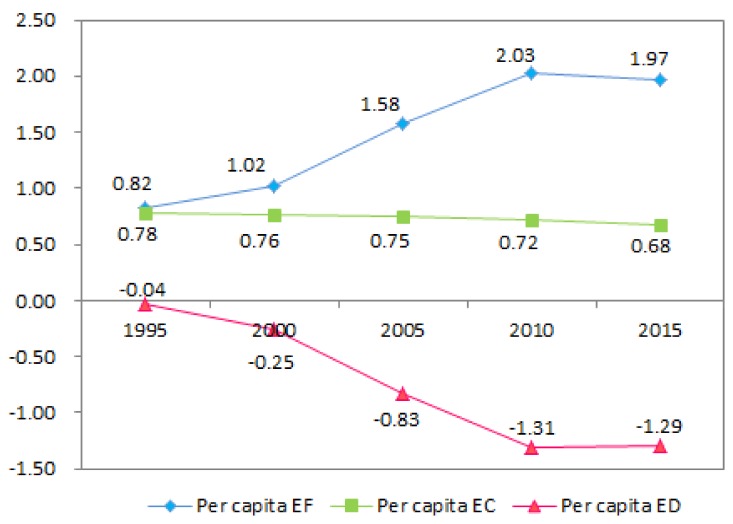
Per capita trends of EF, EC, and ED in northwestern Guangxi from 1995 to 2015.

The per capita EF, EC, and ecological profit and loss in northwestern Guangxi were calculated through the ecological footprint model (Table 6). The available per capita EC is expressed as EC * 0.88 (because 12% of the biodiversity conservation land should be reserved after deduction). Based on this, the change in the overall trend of the EF from 1995 to 2015 was obtained, and the broken line graph of the supply and demand of the EF from 1995 to 2015 is presented in Figure 2.

From Table 6, it can be seen that the per capita EF for northwestern Guangxi increased from 0.8186 hm^2^ in 1995 to 2.0312 hm^2^ in 2010 and decreased to 1.9657 hm^2^ in 2015. For the same period, the available per capita EC decreased from 0.7805 hm^2^ in 1995 to 0.6769 hm^2^ in 2015. From 1995 to 2015, the EF per capita and the available per capita EC for northwestern Guangxi experienced opposite trends. The change in the EC from 1995 to 2015 is generally small, and the changes in the ED are caused mainly by the change in the EF, resulting in the per capita ED increasing from 0.0380 hm^2^ in 1995 to a peak of 1.3065 hm^2^ in 2010 and then falling to 1.2888 hm^2^ in 2015.

The results show that the demand for natural resources in northwestern Guangxi gradually increased from 1995 to 2015, which increased the positive impact on the ecological environment. This result is also in line with the fact that the northwestern Guangxi economy is growing, and the consumption level of residents is increasing. Therefore, the growth of the EF in northwestern Guangxi may be greatly affected by the increase in per capita consumption level in the region and the development of the industrial economy. The per capita EC has been decreasing. The current ecological protection policy in northwestern Guangxi has no effect, and the ecological environment in northwestern Guangxi cannot always meet the high consumption required for production and by the inhabitants. The ecological deficit has increased in the past 20 years, and the ecosystem of northwestern Guangxi is in an unsustainable development state, which was manifested by the increase in environmental pressure caused by population and industrial development.

### 3.2. Dynamic Evolution of the Per Capita Ecological Footprint and Ecological Carrying Capacity of Karst Counties with Different Poverty Levels in Northwestern Guangxi from 1995 to 2015

**Figure 3:**
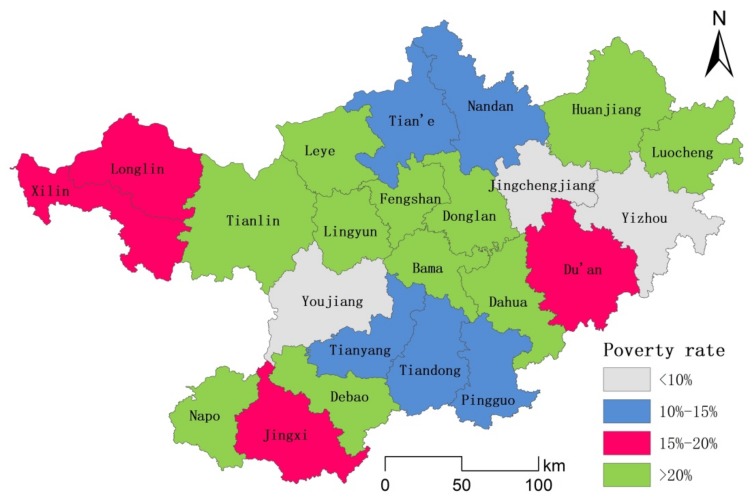
Spatial distribution map of the different types of poverty-stricken counties in northwest Guangxi.

**Figure 4:**
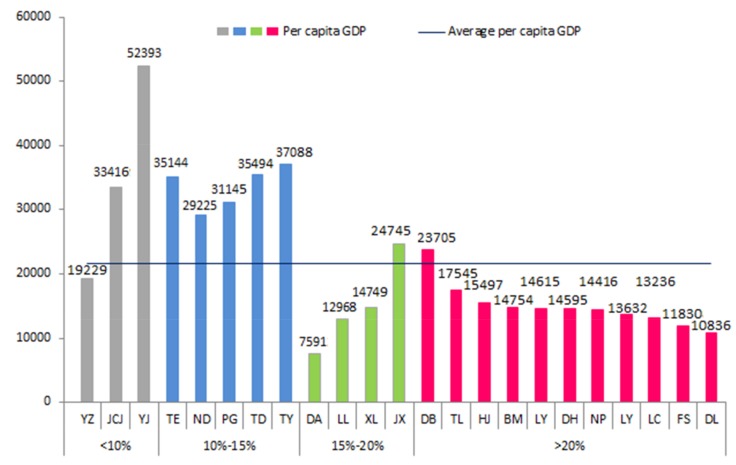
Per capita GDP of poverty-stricken counties in northwestern Guangxi.

According to the poverty rate, northwestern Guangxi is divided into four grades (Figure 3). The specific classification is shown in Figure 3. Among them, Youjiang, Jinchengjiang, and Yizhou belong to the first category, and they have a relatively high economic level and standard of living and a poverty rate below 10%. Except for that of Yizhou, the per capita GDP is much higher than the average GDP (Figure 4). Nandan, Tian’e, Tianyang, Tiandong, and Pingguo belong to the second category, with a poverty rate between 10% and 15%, and a higher per capita GDP than the average GDP level of northwestern Guangxi. The third category included Du’an, Jingxi, Xiling, and Longlin, and was characterized by poor economic development and a poverty rate between 15% and 20%. The per capita GDP of Jingxi is close to the average GDP level of northwestern Guangxi, and the rest are below average. Donglan, Bama, Huanjiang, and the others belong to the fourth category. These areas have the greatest poverty, with the poverty rate exceeding 20%. With the exception of that of Debao, GDP per capita is below average.

**Figure 5:**
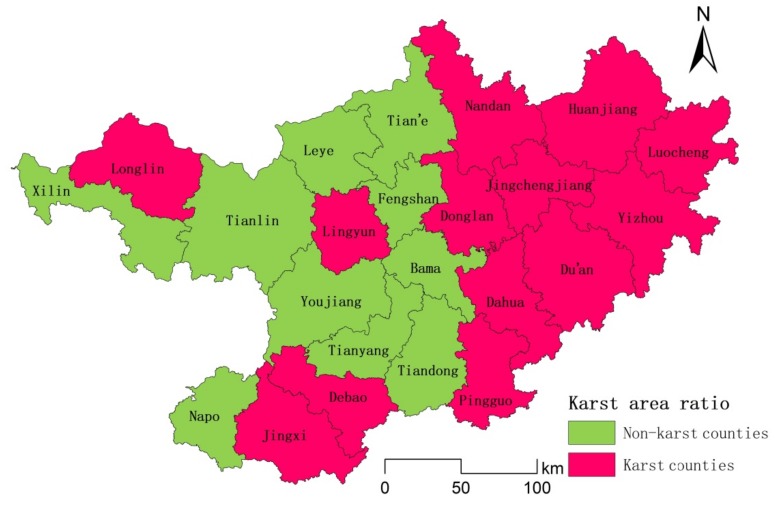
Karst county distribution map of northwestern Guangxi

On the basis of previous studies, we selected counties with a karst area of more than 50% and classified them as karst counties (Figure 5). By combining the karst counties with the per capita GDP and considering the availability of data, we selected three typical poor karst counties in this study (this study mainly considers relatively poor counties, so regions with poverty rates lower than 10% were not considered herein). The typical poverty-stricken karst counties are Nandan County, Du’an County, and Donglan County. We analyzed the characteristics of the evolution of and differences between the EF and EC of typical poverty-stricken karst counties.

#### 3.2.1. Analysis of the Per Capita EF and EC in Typical Counties in 2015

- Analysis of the per capita EF and EC in Nandan County

In 2015, the item with the largest per capita EF in Nandan was arable land (Table 7), while woodland had the highest per capita EC capacity. In terms of ecological profit and loss, the woodlands in Nandan were in an ecologically profitable state in 2015, and the other land use types were in an ED state. In general, the per capita ED of Nandan in 2015 was 0.7569 hm^2^, which is unsustainable.

- Analysis of the per capita EF and EC in Du’an County

In 2015, the item with the largest per capita EF in Du’an was grassland (Table 7), and the per capita EC of woodland was the largest. In terms of per capita ecological profit and loss, the woodland and building land were in an ecologically profitable state. In general, the per capita ED of Du’an in 2015 was 0.3631 hm^2^. This implies that the Du’an County is currently in an unsustainable state.

- Analysis of per capita EF and EC in Donglan County

In 2015, the item with the largest ecological footprint per capita in Donglan County was grassland (Table 7), and the woodland had the highest per capita EC. In terms of profit and loss, only the woodland of Donglan was in an ecologically profitable state. In general, the per capita ED of Donglan in 2015 was 0.1926 hm^2^. This indicates that Donglan was not in a sustainable state in 2015.

- Comparative analysis of the ecological conditions of the counties

**Figure 6:**
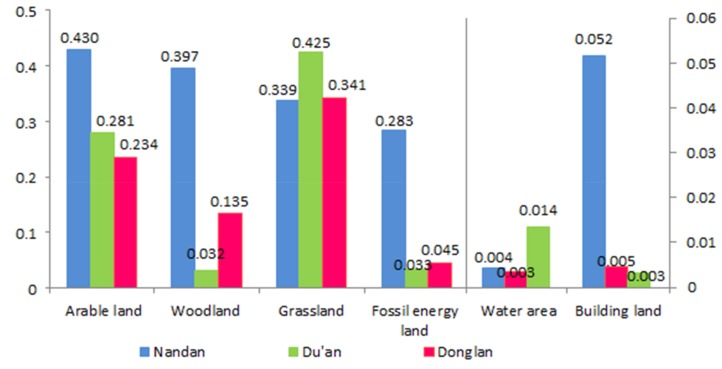
Comparison of the per capita EF of various land types for Nandan, Du’an, and Donglan Counties (2015).

Based on the ecological status of the three counties, a comparative analysis of the per capita EF and EC of each county was conducted (Figure 6). The results indicated that the per capita EF of cultivated land and grassland in the three counties is very high. This is mainly due to the poverty in northwestern Guangxi, the low level of economic development, and the residents’ consumption, which was mainly concentrated on food expenditure, as shown by the relatively high Engel coefficient. In terms of water, Donglan has the largest per capita EF, which was related to the geographical location of Donglan in the middle reaches of the Hongshui River. Nandan has the largest per capita EF in terms of forestland, fossil energy, and building land. This showed that poverty-stricken counties with relatively high levels of economic development have relatively high consumption levels and demand for fossil energy and commercial land.

**Figure 7:**
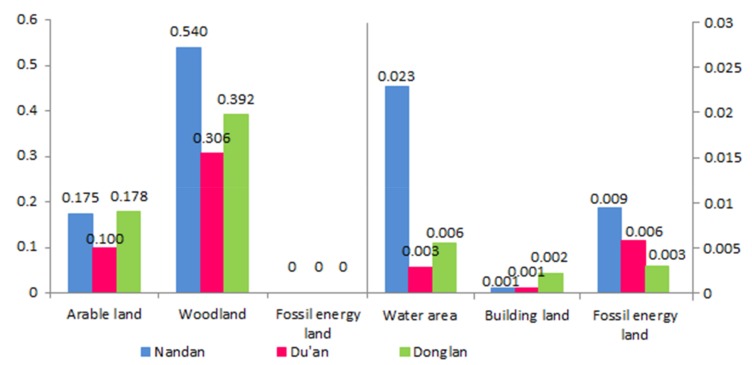
Comparison of the per capita EC of various land types for Nandan, Du’an, and Donglan Counties (2015).

Figure 7 illustrates the per capita EC of various land types in Nandan, Du’an, and Donglan. In terms of cultivated land, Nandan and Donglan have relatively high per capita EC. Due to the relatively low population density and economic development, the local grain production capacity can carry a relatively large population limit. Nandan has the largest per capita EC of forestland. The higher per capita forestland area was the main reason for the relatively high per capita EC of Nandan forestland.

These results imply that the three counties are in an unsustainable state of development, and arable land, grassland, water area, and building land are in a state of deficit, which is consistent with the ED in northwestern Guangxi. This indicates that the use of arable land, grassland, water area, and building land has been generally unreasonable.

#### 3.2.2. Dynamic Evolution of the Per Capita EF and EC in Typical Counties From 1995 to 2015

We used statistical data to establish bioresource and energy consumption accounts and calculate the per capita EF and EC of Nandan, Du’an, and Donglan counties from 1995 to 2015.

- Dynamic evolution of the per capita EF and EC of Nandan County

**Figure 8:**
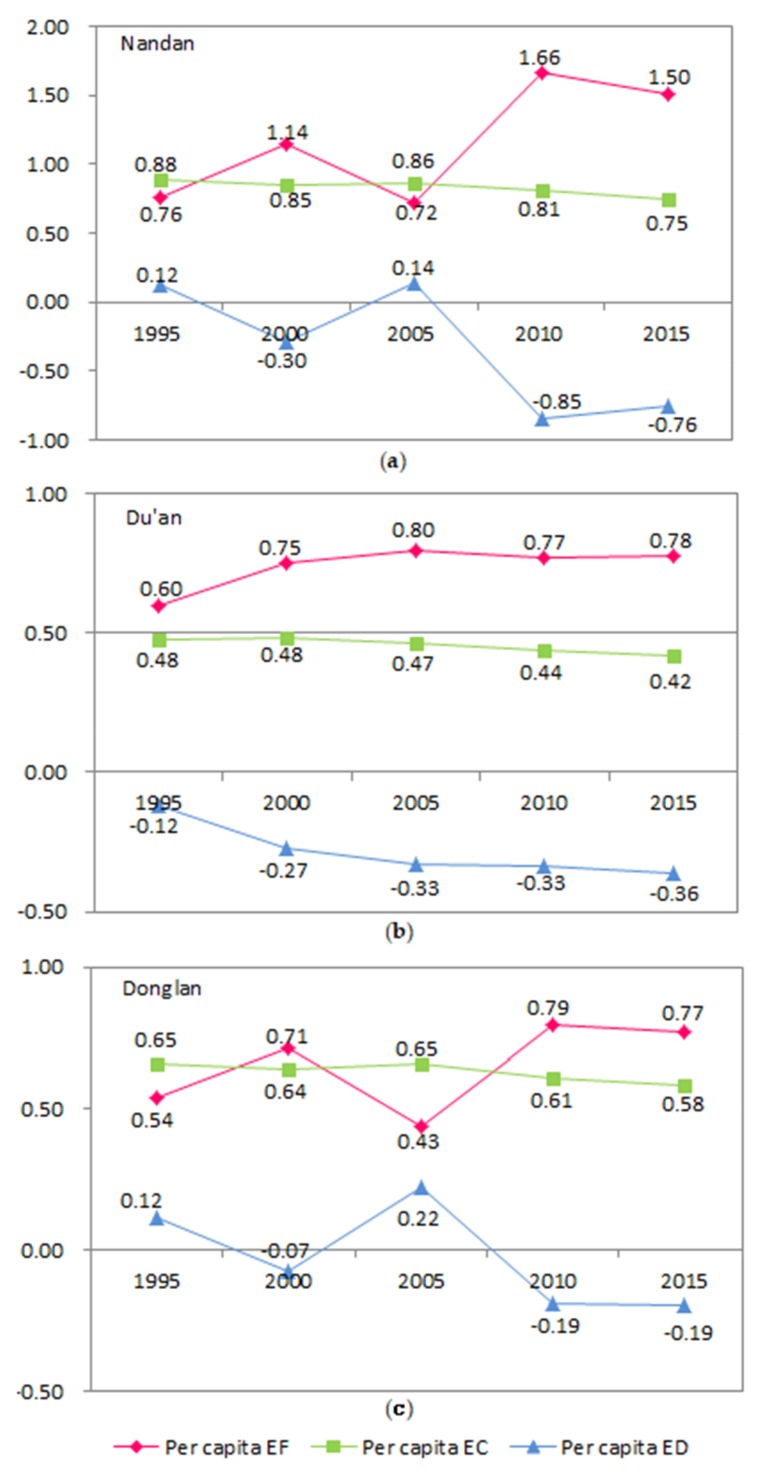
(**a**) Trends of the per capita, EF, EC, and ED of Nandan County; (**b**) trends of the per capita, EF, EC, and ED of Du’an County; (**c**) trends of the per capita, EF, EC, and ED of Donglan County.

Figure 8a illustrates the per capita EF and EC of Nandan County. Overall, the per capita EF shows an upward trend, and the per capita EC and ED show a downward trend. However, the per capita EF decreased slightly in 2005, and the per capita EC and ED increased slightly in 2005. The main reason for this trend is that the total population of Nandan has increased in the past 20 years, but the total population decreased from 2000 to 2005, so the consumption of food has decreased. Nandan has not achieved any results in ecological management in the past 20 years. In the future, we need to take effective measures to improve this situation.

- Dynamic evolution of the per capita EF and EC of Du’an County

Figure 8b depicts an increase in the per capita EF of Du’an from 1995 to 2015, but with little change. In addition, the per capita EC gradually decreases, and the per capita ED fluctuates, but there is an overall reduction. The results show that Du’an is a poor area, the economic development has not been fast in the past 20 years, and the resource consumption has not changed much. This has generated some pressure on the ecological environment.

- Dynamic evolution of the per capita EF and EC of Donglan County

Figure 8c illustrates that the per capita EF of Donglan increased from 1995 to 2015 but decreased in 2005. However, the per capita EC changed slightly from 2000 to 2015 and then slightly increased up until 2005. In addition, the per capita ED increased significantly in 2005 but decreased overall. The main reason for these trends is that the population of Donglan city decreased in 2005 and the consumption of food decreased. The economy of Donglan increased to a certain extent from 1995 to 2015, but the ecological management was poor.

#### 3.2.3. Comparative Analysis of the Ecological Evolution of Nandan, Du’an, and Donglan Counties

By comparing the dynamic evolution of the ecological data from Nandan, Du’an, and Donglan counties, we find that there are significant differences in the ecological development trends of the three counties. The per capita ecological footprint of Nandan County gradually increased from 1995 to 2015, with the highest growth rate reaching 80%, but the growth rate of various indicators decreased from 2010 to 2015. In addition, the per capita ecological deficit of Nandan is the highest among the three counties. The main reason for this is that Nandan has a relatively high level of economic development and the highest per capita GDP, which leads to a relatively high consumption of resources. However, due to the poor overall development of Nandan County and inadequate attention to ecological management, the ecological environment has not been improved, and the ecological carrying capacity is still decreasing. The increase in the ecological footprint per capita is relatively similar in Donglan (which has the highest poverty rate) and Du’an (which has the median poverty rate) at approximately 30%. This is far lower than the increase in the ecological footprint per capita in Nandan County, mainly because of poverty, slow economic development, and the relatively small growth rate of resource consumption in Donglan and Du’an counties. On the other hand, the per capita EF increased gradually from 1995 to 2015 in Donglan County, indicating that Donglan County residents’ demand for resources increased with economic development. However, ecological improvement cannot catch up with the destruction of ecological resources. The ecological deficit of Du’an has been higher than that of Nandan, mainly because Du’an has had not only a low level of economic development but also a high population and a low per capita GDP in the past 20 years. The three per capita indexes (EF, EC, and ED) have not improved significantly in Du’an County. This is mainly due to the slow economic development and stable population growth of Du’an. The per capita GDP of Du’an is the lowest of the three counties, its residents consume a greater amount of resources, and the resources provided by ecological energy have experienced relatively little change.

**Figure 9:**
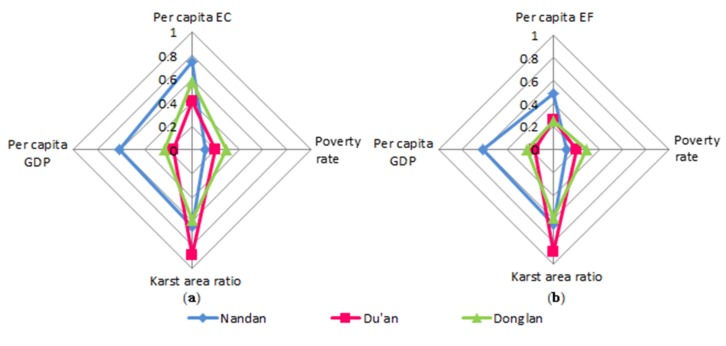
(**a**) Comparison of karst area ratio, poverty rate, per capita GDP, and per capita EC in Nandan County, Du’an County, and Donglan County; (**b**) comparison of karst area ratio, poverty rate, per capita GDP, and per capita EF in Nandan County, Du’an County, and Donglan County.

We conducted a comparative analysis of the relationship between the karst area, poverty rate, per capita GDP, ecological footprint, and ecological carrying capacity of three counties in 2015 (Figure 9). We determined that Du’an has the highest proportion of karst area (reaching 89%), the middle level poverty rate of the three counties, and a middle level ecological footprint. However, the per capita GDP of Du’an is the lowest of the three counties, and the ecological carrying capacity is also the lowest, at only 0.42%. The Donglan karst area accounts for the lowest proportion of the total area (59%), its poverty rate reaches 29%, its ecological footprint is the lowest of the three counties, and its ecological carrying capacity is 0.58 hm^2^, which is the middle level of the three counties. Nandan has the middle proportion of karst area of the three counties (65%), but the per capita GDP is the highest, the poverty rate is the lowest, and the ecological footprint and ecological carrying capacity are the highest.

## 4. Conclusions

### 4.1. Conclusions and Discussion

Based on the ecological footprint theory, this study evaluated the changes in the ecological footprint and ecological carrying capacity in karst areas of northwestern Guangxi from 1995 to 2015. We found that in the past two decades, the per capita ecological footprint of karst areas in northwestern Guangxi has maintained an overall growth trend. In 2015, the per capita ecological footprint of fossil energy land in the karst area of northwestern Guangxi was the largest. However, in the past 20 years, the per capita ecological carrying capacity of karst areas in northwestern Guangxi has not changed much, and the change in the per capita ecological deficit was mainly caused by the very large change in the ecological footprint. On the whole, the contradiction between ecological supply and ecological demand in the karst areas of northwestern Guangxi is large, the development is not sustainable, and the economy and ecological environment are facing great pressure.

We also found that the evolutionary rules of and factors influencing the ecological footprint and ecological carrying capacity of karst counties with different poverty levels are different. In general, from 1995 to 2015, the ecological environment of Nandan, Du’an, and Donglan was in an unsustainable state. The change in the ecological footprint of these three poor karst counties is greatly affected by the level of economic development, and the growth rate of the ecological footprint of counties with high poverty rates is relatively slow. In addition, these three counties are fragile karst counties. Due to the underdevelopment and lack of good ecological environmental management measures, with the increase in demand, the ecological carrying capacity of the three counties has decreased in the past 20 years. In 2015, Du’an County had the highest proportion of karst area and the lowest ecological carrying capacity, but its ecological footprint was not the lowest. Donglan County had the lowest proportion of karst area and the lowest ecological footprint but not the lowest ecological carrying capacity. Karst geological and geomorphic factors, poverty rate, per capita GDP, ecological footprint, and ecological carrying capacity are not simply linearly related. The ecological footprint and ecological carrying capacity are jointly affected by many factors in karst areas of northwestern Guangxi.

Generally, when the ecological footprint of karst counties with different levels of poverty in the study area increase, the ecological carrying capacity will be decreased, this is consistent with the overall trend of the region. However, in agreement with previous studies [2], we also conclude that the ecological change trends in different counties within the region are significantly different. However, the related studies in the past were mainly focused on the change of the overall ecological footprint, ecological carrying capacity of the region, and the prediction of the future, they did not pay enough attention to the differences within the region. Through the analysis of regional differences, we can understand the different ecological problems in different regions and propose improvement measures in a targeted manner. In this study, we mainly analyzed the differences in the ecological footprint and ecological carrying capacity of karst counties with different levels of poverty. The internal mechanism of these differences has not been analyzed. This is the research we need to carry out in the future.

In addition, due to the special nature of the karst area in northwest Guangxi, some statistical work within the region has not been carried out sufficiently. If research methods are used that require a large amount of indicator data, such as the ecosystem service tradeoff method, due to the large number of indicators and the large amount of data requirement, the statistical data cannot meet the standard. Some of the other popular research methods, such as land use-based SWAT models, can perform simulation and forecasting well, but this study is mainly based on the evaluation of the current data to achieve the analysis of regional differences. Therefore, this study we finally selected the traditional ecological footprint research methods. Compared with some recent research methods, the ecological footprint model has more data acquisition methods, including statistical data, land and resources survey, and remote sensing interpretation. Moreover, the ecological footprint method has accumulated a lot of research and has a clear theoretical basis, and the results are easy to verify [61]. However, the evaluation results of the ecological footprint method only reflect the current situation of the region, and cannot simulate and predict the future scenarios [62]. In the future, we need to use some popular forecasting models and methods to predict the future ecological changes in the region.

### 4.2. Suggestions

To promote the sustainable development of karst areas in northwestern Guangxi, we should not blindly increase the per capita EC. We should take different measures according to different poverty-stricken counties. Based on our study results, we recommended the following measures.

On the whole, the karst landform in the northwest of Guangxi is developed, the degree of rock desertification is serious, and soil and water loss occurs readily. Therefore, the whole area should be managed based on the protection of the karst ecological environment, effective use of the existing resources, and improved overall ecological carrying capacity. The local government should plan the land use layout according to the characteristics of the karst area in northwestern Guangxi, coordinate the urban and rural construction, realize the conversion of farmland to forestland and grassland, and carry out the control of rocky desertification. The rural populations in karst areas with fragile ecology should be concentrated in cities and towns, and measures such as "ecological migration" should be actively supported.

Although the poverty rate of Du’an County is not the highest of the three counties, its population is large, its per capita GDP is far lower than the regional average GDP, its economic development is slow, and change in its per capita environmental footprint is not obvious. In addition, 89% of Du’an County is karst area, its ecosystem is fragile, and its ecological carrying capacity is low. Therefore, in addition to properly controlling the population size, we should also strengthen the ecological protection and supervision of the karst landform area and reduce the rocky desertification.

As the county with the lowest poverty rate, the per capita GDP of Nandan County is higher than the regional average GDP, and the economic development is relatively fast. Therefore, resource consumption is relatively large. Measures should be taken to improve resource utilization efficiency, reduce fossil energy consumption, and protect cultivated land, grassland, and forest resources.

Donglan County, as the poorest of the three typical counties, has a large number of impoverished people, and its ecological footprint is the lowest; however, its ecological carrying capacity is not the lowest. Therefore, the key is to control the population of impoverished people, improve the level of economic development, and strengthen environmental publicity and residents’ awareness of environmental protection.

## Figures and Tables

**Table 1:** Description of the land types in the ecological footprint (EF) account.

Land Type	Main Application	Equivalence Factor	Yield Factor
Arable land	Provide crops	2.8	0.98
Woodland	Provide forest products	1.1	0.64
Grassland	Provide livestock products	0.5	0.19
Water area	Provide aquatic products	0.2	1.00
Built-up area	Land for human life and construction	2.8	0.98
Fossil energy area	Absorb carbon dioxide released by humans	1.1	0

Note: (1) The global average biocapacity is 1. (2) In reality, people do not set aside land for absorbing carbon dioxide (Peng et al. 2018).

**Table 2:** Biological resources footprint account of northwestern Guangxi in 2015.

Item	Residents’ Consumption (t)	Global Average Production (kg·hm·10^−2^)	Total EF (hm^2^)	Area Per Capita (hm^2^·Person^−1^)	Type of Cultivated Land
Cereal	2,099,256	2744	2,142,098	0.09132	Arable land
Beans	73,373	1856	110,692	0.00472	Arable land
Potatoes	338,577	12,607	75,198	0.00321	Arable land
Oil-bearing crops	29,873	1856	45,067	0.00192	Arable land
Cotton	673	1000	1884	0.00008	Arable land
Fiber crops	195	1500	364	0.00002	Arable land
Sugar cane	7,524,257	18,000	1,170,440	0.04990	Arable land
Tobacco	14,816	1548	26,799	0.00114	Arable land
Cassava	124,841	9849	35,491	0.00151	Arable land
Vegetables	3,638,881	18,000	566,048	0.02413	Arable land
Tea	11,612	566	22,567	0.00245	Woodland
Fruit	1,298,604	18,000	79,359	0.00861	Woodland
Chestnuts	50,217	2280	24,228	0.00263	Woodland
Walnuts	1447	2254	706	0.00008	Woodland
Tung oil seeds	65,899	1856	39,057	0.00424	Woodland
Tea oil seeds	81,887	1856	48,532	0.00527	Woodland
Turpentine	30,339	3900	8557	0.00093	Woodland
Dry bamboo shoots	6028	945	7017	0.00076	Woodland
Star anise	35,034	945	40,780	0.00443	Woodland
Cinnamon	100	945	116	0.00001	Woodland
Fennel oil	2779	253.61	12,054	0.00131	Woodland
Pork	325,175	74	2,197,128	0.52454	Grassland
Beef	42,793	33	648,379	0.15479	Grassland
Lamb	22,088	33	334,667	0.07990	Grassland
Poultry	98,733	457	108,023	0.02579	Grassland
Eggs	10,344	400	12,930	0.00309	Grassland
Milk	306	502	305	0.00007	Grassland
Honey	789	50	7890	0.00188	Grassland
Silkworm cocoons	118,093	1000	59,047	0.01410	Grassland
Aquatic products	224,478	258	174,014	0.10386	Water area
Wood cutting amount	425.58	1.99	2,352,452	0.25528	Woodland
Bamboo cutting amount	240,328.4	3506.379	75,394	0.00818	Woodland

**Table 3:** Fossil energy footprint account of northwestern Guangxi in 2015.

Item	Total Consumption (t)	Conversion Factor (GJ·t^−1^)	Global Average EF (GJ·hm^−2^)	Total EF (hm^2^)	Area Per Capita (hm^2^·Person^−1^)	Land Type
Coal	11,959,600	20.934	55	5,007,245	0.5434	Fossil energy land
Crude oil	887,200	41.868	93	439,353	0.0477	
Diesel oil	642,200	42.705	93	324,384	0.0352	
Liquefied petroleum gas	45,900	50.2	71	35,699	0.0039	
Electricity	2,090,400 ^1^	0.0036 ^2^	1000	210,712	0.0090	Building land

^1^ The unit of power consumption is 104 kW·h; ^2^ The unit of the conversion coefficient of power is GJ·hW^−1^·h.

**Table 4:** The EF in northwestern Guangxi in 2015.

Land Type	Area Per Capita (hm^2^·Person^−1^)	Equivalence Factor	Per Capita EF (hm^2^·Person^−1^)
Arable land	0.1780	2.8	0.5010
Woodland	0.2942	1.1	0.3236
Grassland	0.8042	0.5	0.4021
Water area	0.1039	0.2	0.0208
Building land	0.0090	2.8	0.0252
Fossil energy land	0.6301	1.1	0.6931
Total	2.0193		1.9657

**Table 5:** The ecological carrying capacity (EC) in northwestern Guangxi in 2015

Land Type	Area (hm^2^)	Equivalence Factor	Yield Factor	Total EC (hm^2^)	Per Capita EC (hm^2^·Person^−1^)
Arable land	950,651	2.8	0.98	2,295,556	0.3114
Woodland	5,184,605	1.1	0.64	3,211,967	0.4357
Grassland	735,246	0.5	0.19	61,467	0.0083
Water area	52,332	0.2	1.00	9210	0.0012
Building land	38,182	2.8	0.98	92,199	0.0125
CO_2_ absorption land	0	1.1	0	0	0
Total	6,961,016			5,670,398	0.7692

**Table 6:** Trends in the per capita EF, EC, and ecological deficit (ED) in northwestern Guangxi from 1995 to 2015.

Year	Per Capita EF (hm^2^·Person^−1^)	Per Capita EC (hm^2^·Person^−1^)	Available Per Capita EC (hm^2^·Person^−1^)	Per Capita ED (hm^2^·Person^−1^)
1995	0.8186	0.8870	0.7805	−0.0380
2000	1.0151	0.8666	0.7626	−0.2525
2005	1.5787	0.8514	0.7492	−0.8295
2010	2.0312	0.8235	0.7247	−1.3065
2015	1.9657	0.7692	0.6769	−1.2888

**Table 7:** Summary of the per capita EF, EC, and ED of Nandan, Du’an, and Donglan Counties (2015).

Item	Per Capita EF (Global Standard Area)			Per Capita EC			Per Capita ED		
	Nan Dan	Du’an	Dong Lan	Nan Dan	Du’an	Dong Lan	Nan Dan	Du’an	Dong Lan
Arable land	0.4295	0.2808	0.2344	0.1748	0.1000	0.1778	−0.2547	−0.1808	−0.0566
Woodland	0.3968	0.0316	0.1353	0.5398	0.3060	0.3918	0.1430	0.2744	0.2565
Grassland	0.3389	0.4248	0.3414	0.0229	0.0029	0.0056	−0.3160	−0.4218	−0.3358
Water area	0.0044	0.0033	0.0135	0.0006	0.0006	0.0022	−0.0038	−0.0028	−0.0113
Building land	0.0516	0.0045	0.0033	0.0095	0.0058	0.0030	−0.0422	0.0013	−0.0003
Fossil energy land	0.2832	0.0334	0.0451	0	0	0	−0.2832	−0.0334	−0.0451
Sum	1.5044	0.7784	0.7730	0.7476	0.4153	0.5804	−0.7569	−0.3631	−0.1926
